# Contribution of Zinc and Zinc Transporters in the Pathogenesis of Inflammatory Bowel Diseases

**DOI:** 10.1155/2019/8396878

**Published:** 2019-03-10

**Authors:** Wakana Ohashi, Toshiyuki Fukada

**Affiliations:** ^1^Department of Molecular and Medical Pharmacology, Graduate School of Medicine and Pharmaceutical Sciences for Research, University of Toyama, Toyama, Japan; ^2^Molecular and Cellular Physiology, Faculty of Pharmaceutical Sciences, Tokushima Bunri University, Yamashiro, Tokushima, Japan

## Abstract

Intestinal epithelial cells cover the surface of the intestinal tract. The cells are important for preserving the integrity of the mucosal barriers to protect the host from luminal antigens and pathogens. The mucosal barriers are maintained by the continuous and rapid self-renewal of intestinal epithelial cells. Defects in the self-renewal of these cells are associated with gastrointestinal diseases, including inflammatory bowel diseases and diarrhea. Zinc is an essential trace element for living organisms, and zinc deficiency is closely linked to the impaired mucosal integrity. Recent evidence has shown that zinc transporters contribute to the barrier function of intestinal epithelial cells. In this review, we describe the recent advances in understanding the role of zinc and zinc transporters in the barrier function and homeostasis of intestinal epithelial cells.

## 1. Introduction

The gastrointestinal tract absorbs nutrients from digested foods and protects against luminal antigens and invading pathogens, including commensal bacteria and food. For this protective function, the intestines have developed a robust system of physical, chemical, and biological mucosal barriers. Defects in the mucosal barriers lead to the translocation of luminal antigens into the host, which induces host immune and inflammatory responses. These responses increase the susceptibility to various gastrointestinal diseases. Maintenance of the mucosal barrier system is therefore a critical issue in intestinal health [1]. The intestinal epithelial cells generate and maintain the mucosal barriers by continuous renewal [2]. Any impairment in the renewal cycle perturbs the mucosal barriers. Maintenance of the intestinal epithelial homeostasis is essential for preserving mucosal barrier functions.

Zinc is an essential trace element for all living organisms and is involved in a variety of important biological processes. It is a nonredox transition metal that serves as a catalytic cofactor and has structural functions in numerous proteins. Bioinformatics analyses have revealed that approximately 10% and 6% of the genes in the genomes of humans and bacteria, respectively, encode products with zinc-binding potential [3–5]. The functions of zinc-binding proteins are highly divergent and include transcription factors, DNA synthase, ubiquitin ligase, receptors, and kinases [3]. Zinc acts as a signaling molecule, such as second messengers, to mediate signaling pathways [6–9]. Zinc deficiency dysregulates cellular functions. Excess zinc is also toxic to cells. Thus, the level and distribution of zinc must be tightly fine-tuned. Zinc transporters regulate the distribution of zinc by controlling zinc influx and efflux via organelle membranes, thereby contributing to the maintenance of zinc homeostasis [10, 11]. Zinc transporters consist of two families: solute carrier (SLC) 39A and SLC30. The SLC39A/Zrt- and Irt-related protein (ZIP) family has 14 members and functions to transport zinc from the extracellular/organelle region into the cytosol. The SCL30A/Zn transporter (ZnT) is composed of 10 members and participates in exporting zinc from the cytosol. By regulating the flux of zinc, zinc transporters are involved in the regulation of various zinc-mediated biological functions. The physiological and pathological functions of zinc transporters have been explored through genetic approaches [10–12].

Several lines of evidence suggest that zinc deficiency causes diarrhea and mucosal barrier dysfunction, while zinc supplementation improves symptoms [13]. Thus, in the intestine, zinc is essential to maintain intestinal homeostasis and regulate intestinal disorder. In this review, we focus on the roles of zinc and zinc transporters in intestinal epithelial homeostasis and disorder.

## 2. Intestinal Epithelial Cell: A Critical Player in the Mucosal Barriers

The small intestine is composed of villi that protrude into the lumen and crypts that penetrate the mucosa. In contrast, the large intestines have no protruding villi, consisting only of crypts that allow water absorption. The surface of the intestinal mucosa is covered by a monolayer of intestinal epithelial cells. These cells consist of differentiated and undifferentiated cells [14]. Differentiated epithelial cells include absorptive enterocytes, mucin-producing goblet cells, and Paneth cells, which secrete antibacterial factors and constitute a niche for intestinal stem cells [15–19]. All intestinal epithelial linages are derived from intestinal stem cells. Intestinal stem cells that feature leucine-rich repeat-containing G-protein-coupled receptor 5 (Lgr5) as a marker protein constantly self-renew and produce daughter cells that are designated transit-amplifying (TA) cells [20]. TA cells undergo vigorous proliferation to increase the number of the cells, which differentiate into specialized epithelial lineages. Most differentiated epithelial cells migrate from the crypts to the tip of the villi as they differentiate. Intestinal epithelial cells reaching the tips of the villi undergo apoptosis. The renewal of intestinal epithelial cells takes 3-5 days in mice or 1 week in humans [21, 22].

Intestinal stem cells are cells that are capable of both self-renewal and multipotency. The number of the intestinal stem cells is dynamically regulated. In the steady state, the stem cell number is maintained at a certain level. During development or tissue repair, the stem cells actively self-renew, contributing to increased tissue size or repair [23]. Crypt base columnar (CBC) cells are distributed between Paneth cells at the crypt base and express Lgr5. *Lgr5* is the target gene of canonical Wingless-Int (Wnt) signaling, wherein Lgr5 functions as the receptor and mediates R-spondin signaling, thus potentiating Wnt signaling [24–26]. The Lgr5^+^ CBC cells represent actively cycling stem cells that divide every 24 h [21]. The cells that reside at the +4 position immediately above the Paneth cells in the crypts also represent a stem cell population. The +4 cells dominantly express Hopx and display the slow cycling and DNA label-retention characteristics of quiescent stem cells. Recent studies suggest that they are a reverse stem cell population that can be rapidly recruited to maintain epithelial homeostasis following injury [27, 28]. Indeed, when Lgr5^+^ stem cells are lost, Hopx^+^ quiescent stem cells express Lgr5, leading to their active self-renewal, thereby compensating for the loss of Lgr5^+^ stem cells. Furthermore, Hopx^+^ quiescent stem cells and Lgr5^+^ active cells can compensate for one another when the other cell type is depleted [27].

Goblet cells are a secretory lineage that has an important role in the generation of the mucus layer by secreting O-glycosylated mucin proteins [15]. Mucus gel layers on the intestinal surface present a physicochemical barrier that prevents the invasion of microorganisms [29]. The mucus layer is composed of an outer layer colonized by microorganisms, and a sterile dense inner layer firmly attached to the epithelial surface that contains antibacterial molecules [29]. The mucus layer also contains immunoglobulin A (IgA) produced by B cells in the lamina propria. Secreted IgA in the lumen has a role in neutralizing invading pathogens, antigen presentation to microfold cells (M cells) on the follicular-associated epithelium surface, and disturbance of the attachment of microorganisms on the epithelial surface [30]. In addition to the barrier function, the mucus layer also serves as a nutrient source for intestinal microbes [31]. Polymerized MUC2 in the inner layer is proteolytically degraded to generate the outer layer that is colonized by microorganisms. The mucus layer is highly glycosylated and can be utilized as an energy source by intestinal microbes during dietary fiber-depleted conditions. This leads to the erosion of the mucus barrier, which induces intestinal inflammation [31], implying that the nutritional state could influence the strength of the mucus barrier.

Paneth cells secrete various antibacterial molecules, such as *α*-defensins and RegIII-*γ*, and participate in innate immunity in the gastrointestinal tract [32, 33]. *α*-Defensins are cationic peptides consisting of 18-45 amino acid residues. Upon binding to the cellular surface of a microorganism, *α*-defensins form cationic pores in the membrane. Disruption of the cell membrane integrity leads to an efflux of nutrients or ions, which kills the bacteria [34]. The secretion of antimicrobial peptides depends on bacterial sensing [32]. Paneth cells express pathogen recognition receptors (PRRs), including Toll-like receptors and nucleotide oligomerization domain 2 (NOD2). Therefore, Paneth cells play a role in innate immunity by sensing bacteria and bacterial antigens. Paneth cells also store a large amount of zinc in their granules [35, 36]. Bacteria or bacterial components bind to PRRs, inducing the secretion of antibacterial molecules and zinc from Paneth cells [32, 33, 37, 38]. Additionally, Paneth cells also secrete maintenance factors, such as epidermal growth factors (EGFs) and Wnt3a, to form a niche for intestinal stem cells [39]. Thus, Paneth cells contribute to intestinal stem cell homeostasis and innate immunity by serving as biological barriers.

Cell junctions, which include the tight and adherens junction, firmly link intestinal epithelial cells, thereby forming a physical barrier that inhibits microbial invasion by the paracellular pathway. Pathogen-associated molecular pattern signaling regulates physical barrier functions. TLR2 signaling can enhance intestinal integrity by preserving the expression of zonula occludins-1 (ZO-1), a critical component of the tight junction in the intestinal epithelial cells [40]. The NOD-like receptor family is a component of inflammasomes. In the intestines, molecules that include adenosine triphosphate (ATP), DNA, and RNA are secreted from injured cells. These secreted molecules act as danger signals that result in the activation of inflammasomes. It has been reported that NLRP3- or NLRP6-dependent secretion of interleukin- (IL-) 1*β* and IL-18 contributes to the maintenance of intestinal epithelial homeostasis by promoting the maturation and regeneration of intestinal epithelial cells [41, 42]. Thus, the innate immune signaling regulates both the physiological and biological barrier formations.

## 3. Intestinal Epithelial Cells during Intestinal Inflammation

Emerging evidence suggests that impaired integrity of the mucosal barrier is associated with the pathogenesis of intestinal inflammation [43, 44]. Tight junctions restrict the translocation of luminal materials into the host, thereby protecting the host from luminal antigens and pathogens. Defects in tight junctions (e.g., increased apoptosis of intestinal epithelial cells) cause focal leakages that allow water and small molecules to enter the mucosa, thus inducing a mucosal immunological response [45]. The dysfunction of cell junctions causes intestinal inflammation. Inflammatory bowel diseases (IBDs), which include ulcerative colitis (UC) and Crohn's disease (CD), involve the chronic inflammation of the gastrointestinal tract characterized by epithelial barrier dysfunctions and alterations in immune regulation. IBD patients display increased paracellular permeability with tight junction abnormalities. Moreover, decreased expressions of junctional molecules, such as occludins and claudins, have been found in patients with IBDs [46–48]. Mice lacking claudin-7, an important component of tight junctions, have enhanced paracellular organic solute flux and develop spontaneous colitis [49].

Dysfunctions of Paneth cells have been observed with various intestinal inflammatory diseases. Reduced secretion of *α*-defensin and other antimicrobial peptides was described in patients with CD [50]. In particular, *α*-defensin 5 is downregulated in these patients [51–53]. This might be related to the alteration in epigenetic modification status. The *α*-defensin 5 gene in patients with CD is highly methylated compared to the gene in healthy individuals [54]. Thus, epigenetic programming might be a causal factor in the pathogenesis of CD. NOD2 acts as an intracellular bacterial sensor by recognizing muramyl dipeptide (MDP), a structural component of bacteria. Mice deficient in NOD2 display decreased production of *α*-defensin in Paneth cells and are very susceptible to Listeria infections [37]. Adaptor protein- (AP-) 1B is a polarized sorting factor that mediates the polarized secretion of proteins by regulating intracellular protein sorting. The loss of AP-1B function results in the reduced expression of antimicrobial proteins and impaired IgA secretion, leading to the development of spontaneous chronic colitis by hypoepithelial barrier function [55]. Decreased expressions of AP-1M2 have also been observed in patients with CD [55].

Defective mucin is also involved in the pathogenesis of IBDs. Mucin2 (MUC2) expression is decreased in patients with IBDs [56]. Lack of MUC2 reportedly leads to the development of spontaneous colitis characterized by mucosal thickening and superficial erosions [57]. The same authors reported that mice lacking MUC2 showed severe damage against dextran sodium sulfate- (DSS-) induced experimental colitis.

The pathogenesis of IBDs is multifactorial and includes environmental and genetic factors. Regarding the genetic factors, recent genome-wide association studies (GWAS) have identified multiple genes associated with the risk of IBD [58–61]. Variations in the genes related to epithelial barrier functions, such as *NOD2* and *FUT2*, have been identified as genes associated with the pathogenesis of IBDs [59, 61]. Homeostasis of intestinal epithelial cells is maintained through continuous self-renewal, including rapid proliferation, differentiation, and apoptosis. Wnt/*β*-catenin and Notch signaling have essential roles for the intestinal epithelial turnover [62]. Defects in these processes also lead to the impairment of barrier functions and IBD pathogenesis. GWAS have identified Wnt signaling-related genes T cell factor 4 (*TCF4*) and lipoprotein receptor-related protein 6 (*LRP6*) as associated with the risk for IBD [63]. The functional variant in *LRP6* has been associated with early-onset ileal CD [64]. Furthermore, a recent study reported specific changes in the DNA methylation status in the intestinal epithelial cells that were associated with the development of IBD, suggesting that intestinal epithelial cell functions are epigenetically regulated along with the disease development [65].

## 4. Zinc in Intestinal Diseases

Zinc is an essential trace element in living organisms, including mammalians, bacteria, and plants. It has been estimated that approximately 10% of the human genome encode zinc-binding proteins [3]. Consequently, zinc-binding proteins constitute a large proportion of the total proteome [4]. The functions of these zinc-binding proteins are very diverse. Dysregulation of zinc homeostasis is associated with the pathogenesis of gastrointestinal diseases [13].

Zinc deficiency induces to diarrhea [13, 66, 67]. Reduced serum zinc levels or decreased zinc levels in the colorectal mucosa have been found in patients with persistent diarrhea. Zinc supplementation is effective in the prevention or improvement of diarrhea [67–69]. The World Health Organization has recommended zinc supplementation for the treatment of diarrhea as it reduces the duration and severity of symptoms associated with diarrhea and prevents subsequent episodes [70]. Zinc deficiency can also lead to an increased risk of gastrointestinal infectious diseases [71]. In pathogenic bacterial infections, the tissue zinc level was altered in the inflamed intestine. In a mouse model of Salmonella infection, the level of zinc was reduced in the inflamed gut [72]. Zinc also has a beneficial effect on infectious diseases like shigellosis. Malabsorption of nitrogen and the abnormal loss of mucus and transmucosal protein have been reported in patients with shigellosis. Zinc supplementation improves intestinal barrier function, intestinal permeability, nitrogen absorption, and symptoms and also increases immune responses [73–76].

Serum zinc levels in patients with CD are reduced compared to levels in healthy individuals [13, 77]. The absorption of zinc in patients with IBD is also lower compared to healthy controls. Although the relationship between low serum zinc levels and CD development is somewhat controversial, zinc supplementation appears to have beneficial effects on disease outcomes [78, 79]. A prospective study showed that the supplementation of zinc led to improved outcomes for patients with CD [80]. A cohort study of female IBD patients suggested that zinc intake can reduce the risk for CD [81]. The influence of zinc deficiency on colitis has also been examined in animal studies. For example, zinc deficiency exacerbates the severity of experimental colitis in rats [82, 83]. The severity of colitis has been correlated with serum zinc levels in mice [83]. Consistent with these reports, zinc treatment improved the severity of experimental colitis in mice [84–87]. The molecules that mediate the beneficial effect of zinc in colitis have been explored. GPR39 is a specific zinc receptor expressed on the plasma membrane [88]. Upon sensing extracellular zinc, GPR39 activates downstream signal pathways to regulate cellular functions, including proliferation, differentiation, and survival [89]. GPR39 sensing of extracellular zinc appears to affect the severity of colitis. Mice lacking GPR39 showed increased mortality after the induction of experimental colitis [90].

## 5. Zinc and Zinc Transporters in Intestinal Epithelial Cells

### 5.1. Mucosal Barriers

**Figure 1:**
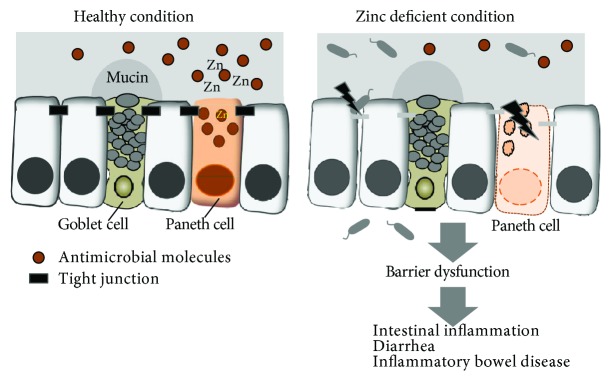
Zinc preserves mucosal barrier functions. In healthy conditions, mucosal barriers, including tight junctions, the mucus layer, and antimicrobial molecules, protect the host from luminal antigens/pathogens. In contrast, in zinc-deficient conditions, mucosal barriers are impaired. Thus, luminal contents can access the mucosal surface and translocate across the epithelial cells, leading to inflammation and intestinal diseases.

**Figure 2:**
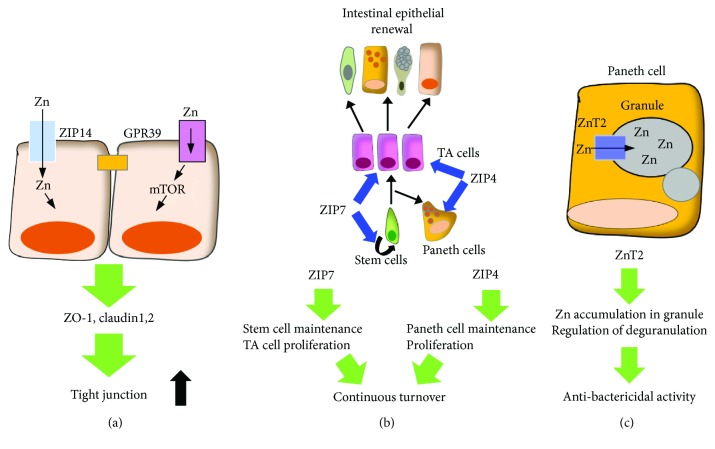
Zinc in intestinal epithelial cells ZIP14 and GPR39 mediates the establishment of the physical barrier through tight junctions (a). ZIP7 and ZIP4 contribute to the maintenance of intestinal homeostasis by securing continuous epithelial turnover (b). ZnT2 facilitates zinc accumulation into the secretory granule, contributing to the antimicrobial function of Paneth cells.

Disruption of the physical barrier formed by intestinal epithelial cells is associated with gastrointestinal diseases [45, 91]. Increased apoptosis of the intestinal epithelial cells causes weakened epithelial barrier tightness and induces focal leaks. Focal leaks lead to increased intestinal permeability and leak fluxes, followed by the activation of immunological responses in the mucosa. Decreased barriers lead to leaky gut properties. Emerging evidence has implicated zinc in the maintenance of the mucosal barriers (Figure 1) [85, 92]. Zinc deficiency leads to reduced expressions of occludin and ZO-1 proteins, resulting in decreased tight junctions in Caco-2 cells [93]. Depletion of zinc induces occludin-3 proteolysis and decreased claudin-3 transcription [94]. In contrast, in mice with bacterial infections, zinc supplementation protects the mice from intestinal dysfunction and intestinal leakage induced by bacterial toxins [95]. Zinc supplementation also enhances tight junctions in Caco-2 cells, characterized by an increase in transepithelial electrical resistance (TEER) and induced expressions of claudin-2, claudin-7, and ZO-1 proteins [96, 97]. Furthermore, zinc facilitates tight junction formation via GPR39 by preserving occludin and ZO-1 expressions in Caco2 and HT29 cells (Figure 2(a)) [98]. Zinc also activates the mammalian target of rapamycin pathway via GPR39 in HT29 cells [98]. Thus, the zinc-GPR39 axis seems to have a regulatory role in tight junction strength between intestinal epithelial cells. The ZIP14 zinc transporter is expressed on plasma membranes and mediates zinc influx into the cytosol, thereby regulating cellular signaling (Figure 2(a)). Mice lacking ZIP14 display increased intestinal permeability associated with altered expressions of tight junction proteins of claudin-1 and claudin-2 [99]. Thus, zinc transporter-mediated zinc signaling may affect the intestinal barrier functions. As described above, Paneth cells contain a large amount of zinc in their granules [35, 36]. Considering that Paneth cells are susceptible to zinc deficiency, zinc seems to contribute to the establishment of a biological barrier, since Paneth cells have a critical role in the production of antimicrobial molecules.

### 5.2. Intestinal Immunity

Intestinal macrophages are abundant in the small and large intestines. In particular, they are found in close proximity to the intestinal epithelial cells [100]. The major functions of intestinal macrophages are sensing, uptake, and clearance of microorganisms invading across the epithelial barrier. Intestinal macrophages thus play an important role in the maintenance of gut homeostasis by contributing to barrier functions. Compared to peritoneal macrophages, intestinal macrophages are highly bactericidal and show increased expressions of metallothionein 1 (MT-1) associated with increased intracellular zinc level [101]. The increased zinc level in intestinal macrophages is missing in mice treated with antibiotics. Furthermore, NOD2 ligand MDPs can induce the upregulation of MT-1 and intracellular zinc levels in intestinal macrophages [101]. Thus, zinc may have a role in regulating the bactericidal potential of intestinal macrophages, thereby mediating a part of symbiotic relationship in the intestine. Dysbiosis has been proposed as a key feature of IBD [102]. There is experimental evidence of the involvement of zinc in the microflora community, whereby excess dietary zinc alters the gut microbiota [103]. Mice fed with an excess zinc diet showed decreased microbial diversity without affecting the bacterial burden. Recently, the association of a variant of zinc transporter *ZIP8* with CD was reported [104]. The healthy *ZIP8* variant carrier showed altered gut microflora partially overlapping with those in patients with CD. Thus, disturbed zinc homeostasis might be associated with dysbiosis.

### 5.3. Intestinal Zinc Absorption

ZIP4 plays an important role in zinc absorption from the small intestine [105]. ZIP4 localized on the apical membrane of intestinal epithelial cells controls the incorporation of luminal zinc into the host by multiple mechanisms [105–108]. The dysfunction of ZIP4-mediated zinc absorption due to *ZIP4* gene mutations causes acrodermatitis enteropathica [109–111]. ZnT1 is detected at the basolateral membrane [112]. Expression of ZnT1 is affected by dietary zinc supplementation. With zinc supplementation, ZnT1 expression is increased in the rat intestine [112, 113]. Since ZnT1 belongs to the SLC30A/ZnT family that mediates zinc export from the cytosol into the extracellular or intracellular compartment, ZnT1 may also mediate zinc transport from the intestinal epithelial cells to the extracellular region (i.e., the blood stream). Thus, ZIP4 and ZnT1 appear to have important roles in the intake of zinc. ZIP5 is localized on the basolateral side of the intestinal epithelial cells and participates in zinc excretion [106, 114]. Mice lacking ZIP5 in intestinal epithelial cells accumulate zinc in the pancreas [115]. Interestingly, expressions of ZIP4 and ZIP5 are oppositely regulated by zinc. In the zinc-deficient condition, expression of ZIP4 is increased and ZIP5 expression is decreased [108]. On the contrary, the ZIP4 level is suppressed in the zinc-adequate condition, whereas ZIP5 expression is induced [108]. Additionally, ZnT5B, which is a splicing variant of ZnT5, has been reported to be localized to the apical membrane of the epithelium in the small intestine [116]. Since ZnT5B is capable of bidirectional transport of zinc [116, 117], ZnT5B appears to mediate zinc influx as well as efflux from the epithelial cells. Thus, zinc absorption and excretion are likely determined by the regulation of specific zinc transporters.

### 5.4. Intestinal Epithelial Homeostasis

Zinc deficiency leads to alterations in the integrity and function of the intestinal epithelial cells, demonstrating a role for zinc in intestinal epithelial homeostasis [118]. However, exactly how zinc carries out this role is unclear. Recent studies have indicated that zinc transporters contribute to the maintenance of intestinal epithelial homeostasis by regulating cellular function.

ZIP4 has a role in the regulation of intestinal epithelial function (Figure 2(b)). Mice lacking ZIP4 in the intestinal epithelial cells have decreased expressions of Sox9, a marker for Paneth cells; induction of mucin, a marker of goblet cells; and loss of zinc in Paneth cells. These findings suggest that ZIP4-mediated zinc incorporation is necessary for the differentiation and maintenance of Paneth cells [119]. Moreover, ZIP4 also contributes to the proliferation of intestinal epithelial cells [119]. Mice lacking ZIP4 display disrupted villus integrity. Thus, ZIP4 appears to be important in preserving intestinal epithelial architecture.

ZnT2 is the zinc transporter expressed on the secretory granules in Paneth cells [120]. ZnT2 promotes the accumulation of zinc in granules in the Paneth cells [120]. Furthermore, ZnT2 regulates the secretion of antimicrobial molecules in Paneth cells (Figure 2(c)) [120]. The authors described degranulated Paneth cells in ZnT2-deficient mice and the unchanged overall structure of the villi-crypt axis. The findings indicate that ZnT2 are not involved in the continuous self-renewal of intestinal epithelial cells, but rather in Paneth cell function [120].

ZIP7 is an endoplasmic reticulum- (ER-) localized zinc transporter that is highly expressed in the crypts, rather than the villi, in the intestine [121]. In the crypts, intestinal stem cells, TA cells, and Paneth cells express ZIP7. A recent study described the essential role for ZIP7 in the maintenance of intestinal epithelial homeostasis (Figure 2(b)) [121]. The authors described that deletion of ZIP7 in the intestinal epithelial cells in mice caused an acute loss of proliferating cells by apoptosis, with the disappearance of intestinal stem cells [121]. Furthermore, ZIP7 deficiency elevated ER stress in TA cells and stem cells [121]. The villus-crypt structure was completely disrupted in the ZIP7-deficient intestine. Additionally, degenerated Paneth cells were found in the ZIP7-deficient crypts, suggesting the essential role of ZIP7 in Paneth cell maintenance [121]. These findings indicate that ZIP7 is indispensable for the rapid turnover of intestinal epithelial cells by preserving the survival of intestinal stem cells, proliferation of crypts cells, and resolving ER stress. Notch, EGF, and Wnt signaling critically contributes to the maintenance of intestinal stem cells as well as proliferation of intestinal epithelial cells [122]. Recent studies have implicated ZIP7 in these signaling events in other cell types. ZIP7 is involved in the activation of extracellular signal-regulated kinases, which is major downstream molecule of EGF in breast cancer cells [123]. ZIP7 participates in the regulation of the trafficking of Notch in T cell acute lymphoblastic leukemia cells [124]. Accordingly, the treatment of ZIP7-binding compound led to the alteration of the zinc level in the ER, disturbed Notch trafficking, and disrupted apoptosis via an ER stress mechanism. Although the relationship between ZIP7 and these signaling events in intestinal epithelial cells remains to be elucidated, ZIP7 may contribute to the maintenance of crypt homeostasis by multiple mechanisms.

## 6. Conclusion

Zinc plays a critical role in the maintenance of intestinal homeostasis by regulating intestinal epithelial cells, host immune cells, and intestinal commensal bacteria. Additionally, recent studies in mice revealed that zinc transporters contribute to regulation of epithelial functions and to the maintenance of intestinal epithelial homeostasis. These findings clarify the self-renewal mechanisms of intestinal epithelial cells as well as the pathogenesis of intestinal diseases. Due to various differences in the features of zinc transporters between mice and humans, the roles of zinc transporters in intestinal epithelial homeostasis and intestinal disease pathogenesis in humans are still undetermined. Further investigations of zinc transporters in humans may advance the understanding of the pathogenesis of gastrointestinal disorders.
